# Low-Cost High-Speed In-Plane Stroboscopic Micro-Motion Analyzer

**DOI:** 10.3390/mi8120351

**Published:** 2017-11-30

**Authors:** Shashank S. Pandey, Aishwaryadev Banerjee, Mohit U. Karkhanis, Carlos H. Mastrangelo

**Affiliations:** Department of Electrical and Computer Engineering, University of Utah, Salt Lake City, UT 84112, USA; devaash88@gmail.com (A.B.); mohit.karkhanis@utah.edu (M.U.K.); carlos.mastrangelo@utah.edu (C.H.M.)

**Keywords:** high speed imaging, stroboscope, vibration mode measurement

## Abstract

Instrumentation for high-speed imaging and laser vibrometry is essential for the understanding and analysis of microstructure dynamics, but commercial instruments are largely unaffordable for most microelectromechanical systems (MEMS) laboratories. We present the implementation of a very low cost in-plane micro motion stroboscopic analyzer that can be directly attached to a conventional probe station. The low-cost analyzer has been used to characterize the harmonic motion of 52.1 kHz resonating comb drive microactuators using ~50 ns pulsed light-emitting diode (LED) stroboscope exposure times, producing sharp and high resolution (~0.5 μm) device images at resonance, which rivals those of several orders of magnitude more expensive systems. This paper details the development of the high-speed stroboscopic imaging system and presents experimental results of motion analysis of example microstructures and a discussion of its operating limits. The system is shown to produce stable stroboscopic LED illumination to freeze device images up to 11 MHz.

## 1. Introduction

Resonating microelectromechanical systems (MEMS) devices have applications in many fields, such as inertial navigation, medical imaging, defense, aviation and avionics, and micro-robotics [1]. Measurements of the device dynamics are essential for the understanding and analysis of vibration eigenmodes. Several dynamic motion measurement techniques have been reported and broadly classified into two categories; (a) real time and (b) time-averaged techniques [2]. Real time techniques, such as Laser Doppler Vibrometry (LDV), have been extensively used over the years [3]. LDV performs the dynamic analysis of the surface under examination by extracting the Doppler frequency shift of a reflected laser beam one spot at a time. The non-contact nature and high precision of this technique makes it attractive for the characterization of MEMS devices. However, the real-time nature of the measurement requires very high-speed recording of measurements and expensive electro-optical systems.

In recent times, optical microscopy coupled with stroboscopic (pulsed) time-averaged imaging has attracted a lot of attention for imaging rigid body vibrations as an alternative to LDV [4,5,6,7,8,9,10,11,12]. Several researchers have reported in-plane, out-of-plane, and full three-dimensional (3D) motion analysis of MEMS structures using stroboscopic imaging coupled with interferometry [4,5]. A major advantage of using pulsed light is that this technique can produce ‘freeze’ images of high speed device motion with low-frame rate digital complementary metal-oxide-semiconductor (CMOS) cameras; hence, it can measure the motion of microstructures that are vibrating at MHz frequencies at a lower cost than LDV. Further use of digital image post processing techniques has also enabled researchers to analyze microstructure motion at very high resolution in the order of a few nanometers [4,8].

Both LDV and stroboscopic MEMS device motion analyzers are commercially available and are capable of analyzing device in-plane motion of up to 20–25 MHz [13,14]. The pulse width for imaging is ~100 ns for the Polytec MSA-400 system (Polytec Inc., Irvine, CA, USA) [13], which in turn limits the maximum velocity of moving microstructures to be imaged as 10 m/s. The stroboscopic imaging system designed by us has a capability of imaging with 50 ns pulse, which is capable of measuring as high as 20 m/s velocity of MEMS structure. The Lyncée Tec system (Lyncée Tec., Lausanne, Switzerland) [14] uses a pulsed laser for stroboscopic imaging and has a 7.5 ns pulse imaging pulse width, which is currently the state of the art system available for in-plane motion imaging, but it also costs upwards of $200,000. These sophisticated and costly instruments however are largely out of reach for most MEMS researchers. In this paper, we have developed a low cost in-plane motion analyzer which can be attached to a conventional probe station. The system is implemented with a combination of off-the shelf microcontrollers and CMOS logic circuits, a low-light monochrome CMOS camera and a high-power lighting LED pulsed light source. The low-cost system is capable of exposure times of ~50 ns, thus enabling real time synchronous resonance motion measurement of high speed microstructures in the MHz region. With this system, sharp images of a resonating comb drive at a velocity ~20 m/s were captured by a CMOS camera with a resolution of ~0.5 μm without the use of any cumbersome post image processing algorithms. Our system is approximately ~98% less expensive than commercially available solutions that are provided by Polytec and Lyncée Tec [13,14].

## 2. Low-Cost Stroboscopy Imaging System

**Figure 1:**
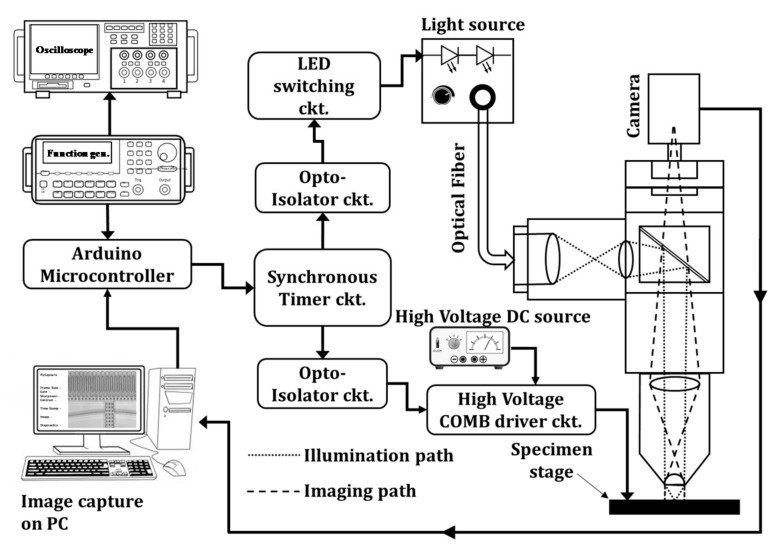
Block diagram of stroboscopic imaging system.

Figure 1 shows the schematic of the low-cost stroboscopic imaging system. The computer controlled system consists of four major blocks: (i) the synchronous timer circuit, (ii) the LED switching circuit, (iii) the high voltage comb driver circuit, and (iv) a conventional probe station imaging microscope with a low-light CMOS digital camera. The imaging system produces a periodic sharp pulse of light from a high-power LED that is synchronized with the device drive, thus producing stationary images of the oscillating device, provided that the device motion is periodic and the period being equal to the ratio of the excitation signal period divided by an integer factor. Stroboscopic systems such as this require precise timing and synchronization of all the signals that are produced by a timer generator circuit.

### 2.1. Timer Generator Circuit

**Figure 2:**
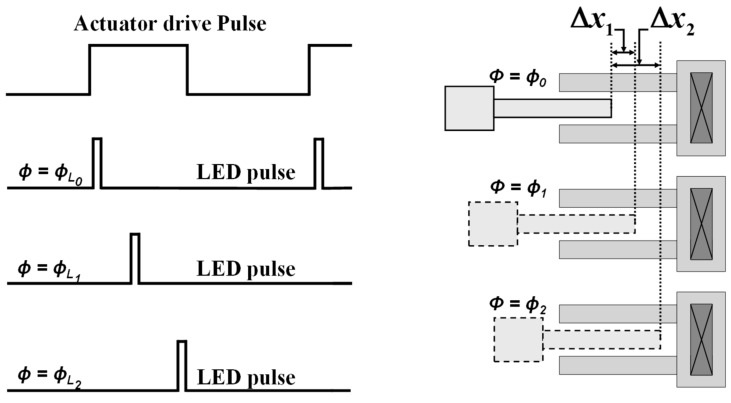
Schematic representation of stroboscopic imaging by phase delayed imaging light-emitting diode (LED) pulse.

This circuit produces all of the synchronized signals that are necessary for driving the device and LED strobe. In time-averaged stroboscopic imaging, the MEMS device is driven by a periodic signal at the desired excitation frequency. The device position at a given phase is captured by the CMOS camera by flashing a very narrow but bright pulse of light with a known delay from the leading edge of the drive signal. Figure 2 illustrates a schematic plot of phase relations between the drive and LED pulses, and the corresponding freeze image for a vibrating comb finger example. Since the motion of the MEMS device is periodic, the recorded image is stationary, and the image brightness that is recorded by the camera is increased simply by integration over many of these pulses. It is also important to note that since the camera integrates the image over the duration of each pulse; the LED pulse should be as sharp as possible to prevent any image smearing.

In order to image the device motion at different times, it is necessary to vary the phase delay *ϕ* of the strobe pulse. The delayed and narrow LED strobe pulse can be generated using different circuit schemes. A common method uses tunable analog delay lines or analog monostable multivibrators. The main disadvantage of analog delay schemes is that the LED pulse phase would change when the drive frequency is swept. This phase however does not change if the LED pulse is generated digitally. For example, one could generate a strobe pulse with a modulo-*M* digital counter every time that the count reaches a particular value *N_L_ < M*. High-precision periodic digital strobe signals can be generated with high-speed finite-state machines using programmable gate arrays or high-speed transistor-transistor logic (TTL) or CMOS logic chips. In this paper, we utilize a TTL implementation due to its simplicity and low cost.

**Figure 3:**
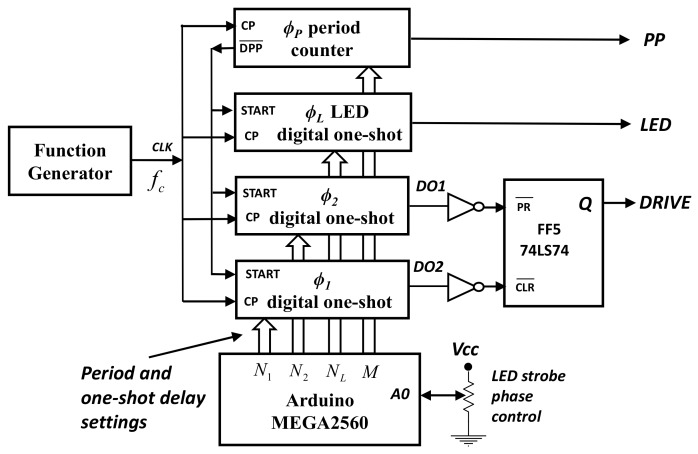
Schematic diagram of the timer circuit showing the generation of the output pulses using the digital delay modules.

Figure 3 shows the schematic of the digital timer generator block diagram. It utilizes two types of digital modules. The first module produces a programmable modulus period signal (*PP*) of frequency *f_c_/M*. The second module is a digital one-shot circuit that produces a single delayed pulse at a specified count *N_i_* starting from the rising edge of the *START* input. For example, the phase delay for such a digital pulse is thus *ϕ_i_* = 2π *N_i_/M* radians.

**Figure 4:**
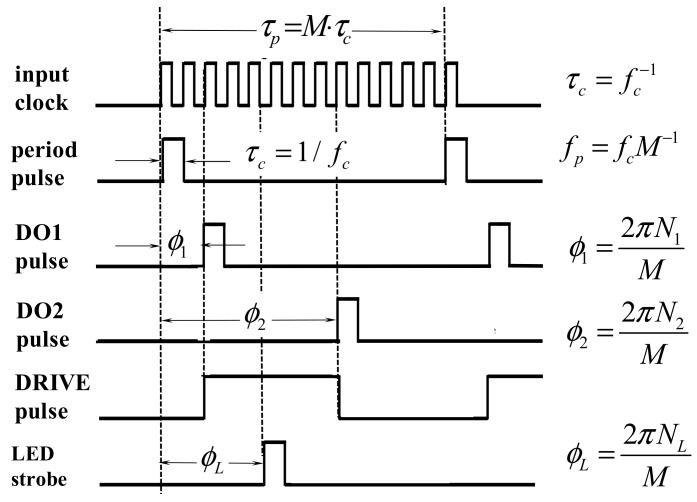
Schematic logic diagram indicating generation of both strobe and drive signals from four narrow digital delay pulses at different digital phase *ϕ_0_*–*ϕ_3_*.

In order to produce the *PP* period, LED pulse and *DRIVE* signals we utilize one period module and three digital one-shot modules. Two of the digital one-shots are used to signal the beginning and end of the *DRIVE* signal within the period *PP*. The value of each *N_i_* is loaded into the period and one-shot modules using an Arduino Mega 2560 microcontroller (Digi-Key Electronics, Thief River Falls, MN, USA). These values are set by the Arduino programming code and the digitized voltage at the Arduino input A_0_ that is provided by a phase control potentiometer. Figure 4 shows the timing diagrams of the resulting digital timing signals.

**Figure 5:**
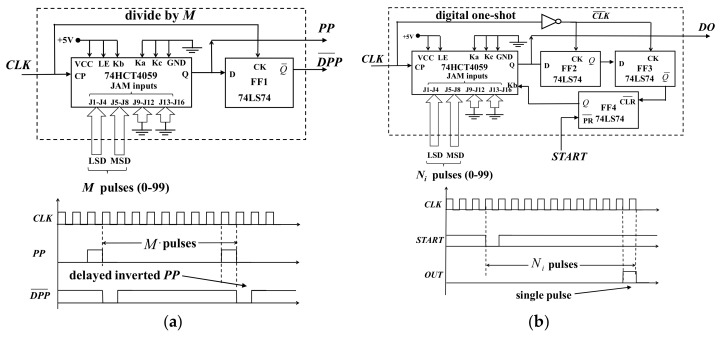
(**a**) Schematic and timing diagram of the period module used as period pulse generator. The period module produces a repeating pulse every *N_i_* input pulses. (**b**) Schematic and timing diagram of digital one-shot module. The digital one-shot produces a single delayed pulse after the *START* signal goes high.

Figure 5a shows the implementation of the period counter module using a 74HCT4059 CMOS programmable divide-by-n counter and ½ of a 74LS74 D flip-flop. The counter chip first loads a two-digit binary-coded decimal (BCD) digital number between 0 and 99 at the counter J1–J8 JAM inputs representing the number of clock pulses and the period length. Then it subsequently counts down to zero and produces an output pulse (*PP*). The counter next loads the BCD number again, and repeats the cycle. A digitally delayed and inverted period pulse ($\bar{DPP}$) is also generated through the D flip-flop.

Figure 5b shows the digital one-shot module. This module has two inputs, a clock pulse from the function generator and a *START* pulse, which is same as the $\bar{DPP}$ output pulse from the period counter. The one-shot module produces a single pulse delayed at (*N_D_*) < *M* that is triggered by a high logic level at the *START* input. The one-shot consists of a 74HCT4059 counter [15] (NXP Semiconductors, Eindhoven, Netherlands), 2D flip-flops and 1 RS flip-flop. The down counter is configured such that the counter initial value *N_D_* is first loaded while the *k_b_* control pin is at logic low. The counter remains in the loading state until the state of FF4 is changed. When the *START* signal goes to logic low, it presets FF4 and the *k_b_* control pin goes to logic high and the counter starts counting down. When the count reaches zero its *Q* output produces a *DD* pulse, which is subsequently delayed by FF2 and FF3 and used to clear flip-flop FF4 setting the counter loading mode until a new *START* signal again goes to logic low, restarting the single-shot cycle.

### 2.2. High Voltage MEMS and LED Driver Circuits

**Figure 6:**
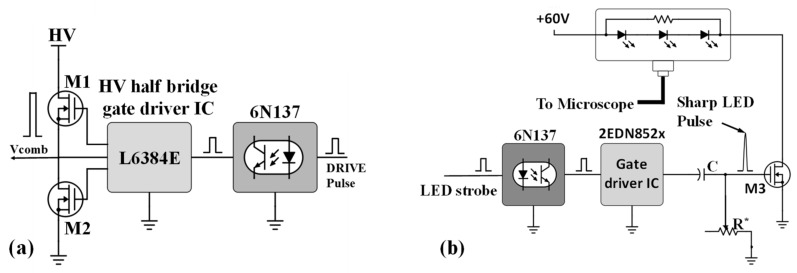
(**a**) Schematic of the half bridge gate driver configuration of high voltage MEMS driver ckt. and (**b**) schematic of the high-speed LED switching ckt. using an ultrafast gate driver integrated circuit (IC) with an R*C differentiator ckt. for producing sharp LED pulses.

These circuits convert the low voltage digital signal from the timer circuit to a high voltage and high-power signals that are needed to drive the MEMS devices and the LED. Figure 6a shows a schematic of the MEMS and LED driver circuits. High voltage signals needed to drive electrostatic MEMS actuators are generated using a L6384E (STMicroelectronics, Geneva, Switzerland) half bridge gate driver chip connected to two high speed, high voltage power metal-oxide-semiconductor field-effect transistor (MOSFETs) (STF2N80K5, STMicroelectronics, Geneva, Switzerland) M1 and M2. This configuration enables the output to switch from 0 to a specified high voltage. The input of the half-bridge is connected to the TTL timer circuit through an optoisolator (6N137, Lite-On, Taipei, Taiwan) which prevents any high voltage back feed onto the timer circuit. The half bridge driver circuit works up to a maximum frequency of about 0.5 MHz.

Figure 6b shows the LED pulse generation circuit. The LED signal from the timing generator drives an optoisolator (6N137, Lite-On, Taipei, Taiwan) and a high-current power MOSFET driver chip (2EDN852x, Infineon Technologies, El Segundo, CA, USA). This driver chip can produce a peak transient gate currents of 1.5 A that is necessary for high speed MOSFET switching (~20 ns). A high-lumen lighting LED (CXM-32 series high lumen LED from Luminus (Sunnyvale, CA, USA) connected to the drain of M3 is connected to an illumination fiber is used to provide pulsed light. The duration of LED light pulse width is controlled by an R*C differentiator circuit connected to the gate of M3 which makes the pulse sharper to ~50–80 ns.

### 2.3. Imaging Optics

The LED generated a cool white light that was centered at a wavelength λ = 450 nm with an output flux of 18,820 lumens. The strobed LED light is introduced into an epi-illuminated microscope (Mitutoyo WF series) with a Mitutoyo Plan Apo infinity corrected long working distance (WD) objective. The magnification on the lens objective was 20x with a numerical aperture (NA) of 0.42. Optical resolution for this set-up can then be estimated by using Abbe’s limit (*R = 0.5λ/NA*), which gives a resolution of ~0.5 μm. Any motion below this value cannot be observed optically and requires image post processing. The freeze frame image is recorded by a c-mount 14-bit 2.8 MP monochromatic Grasshopper 3 series camera from FLIR Integrated Imaging Solutions Inc. (Wilsonville, OR, USA) with a pixel size of 3.69 µm × 3.69 µm. The high quantum efficiency and low temporal dark noise level on this camera sensor generates an image with high signal to noise ratio. In order to obtain a faithful image from the camera, at least three pixels need to be excited i.e., an image size of ~11 µm on the sensor which back annotated to the device under test (DUT) for a 20x magnification objective means that a movement of ~0.55 µm can be sensed by the camera. Since this value is about the same as the optical resolution of the microscope objective, the camera itself does not put any constraint on the image resolution. As high as 26 frames per second (FPS) can be measured with this camera. For stroboscopic freeze-frame imaging system where capturing a static image does not require a high FPS, instead, using a low frame rate (3 FPS) increases the image brightness. The real-time image from the camera is displayed on a PC using the FlyCapture software interface from FLIR Integrated Imaging Solutions Inc. (Wilsonville, OR, USA). The total cost of the timer generator, MEMS and LED drive circuits and imaging system excluding the microscope and external signal generators was <$2000 USD.

## 3. Test Device Fabrication

**Figure 7:**
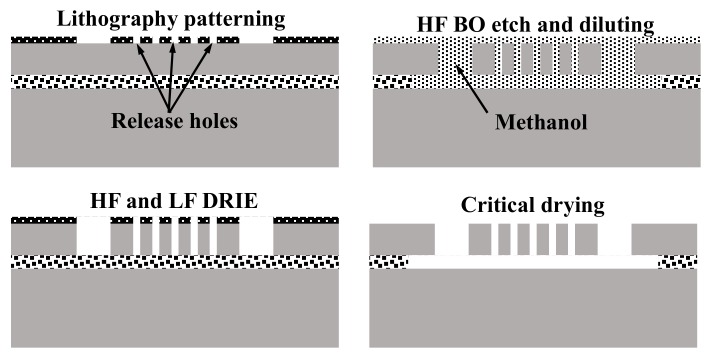
Simplified fabrication process of test comb drive microstructures.

**Figure 8:**
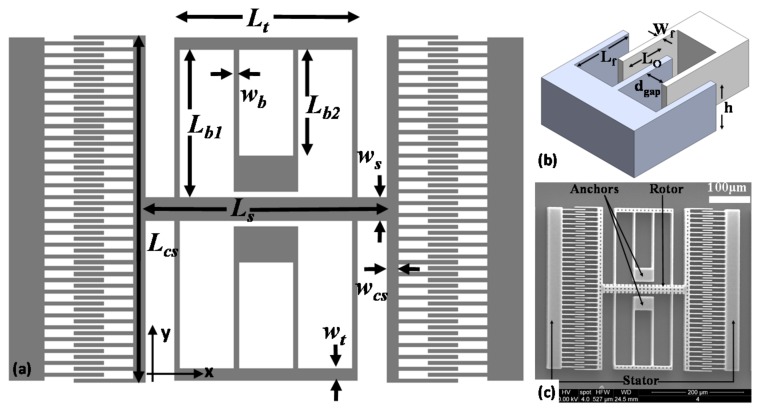
(**a**) Two-dimensional (2D) schematic of the COMB drive actuator with critical dimensions labeled. (**b**) 3D schematic of the COMB drive actuator with the rest of the critical dimensions labeled and (**c**) SEM photographs of the released COMB drive microactuator used for testing.

We have used electrostatic comb drive devices for testing the stroboscopic imaging system. Figure 7 shows a simplified fabrication scheme for the comb drive microactuators. Devices were fabricated on a silicon on insulator (SOI) wafer with 30 μm device layer and 1 μm buried oxide using a single mask process. The comb features were etched on the wafer through High Frequency deep reactive ion etching (DRIE) for first 25 μm ending with a low frequency etch for the last 5 μm to avoid any footing at the buried oxide layer, using positive photoresist as etch mask. Following the DRIE etching, the comb drives were released by etching the buried oxide (BO) in a 5:1 BHF bath. Etched release holes on the wide features, such as main shuttle and trusses, enabled a faster release to prevent anchors from coming off. The HF dip was then diluted with a large quantity of deionized (DI) water. The DI water is then replaced with methanol by dilution. All of this while the die is kept inside the solution in order to prevent stiction of the released structures with the substrate underneath. Device dies were then supercritically dried. Figure 8a,b shows the schematic of the comb drive actuator with all of the important dimensions and Figure 8c shows the SEM images of the released devices.

## 4. Comb Drive Motion

**Figure 9:**
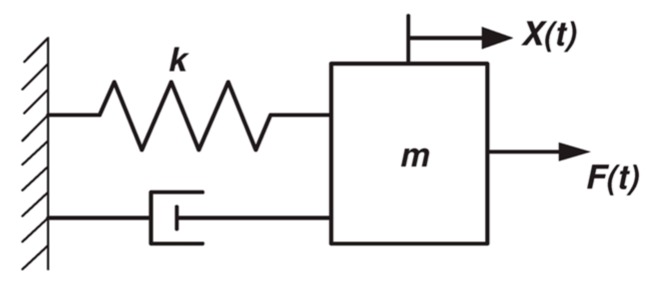
Spring mass damper lumped model equivalent of the comb drive actuators.

The motion of the comb drive is governed by the second order differential equation for forced oscillations of a spring mass damper system, as given by the Equation (1) and shown in Figure 9.

(1) $$m_{eff}\ddot{x}+b\dot{x}+k_{eff}x=F_{ext}(t,\omega )$$

where *m_eff_* is the effective mass of the system under motion, *b* is the damping coefficient, *k* is the net stiffness constant for the folded beam system, *F_ext_* is the electrostatic excitation force which is a function of time (*t*) and frequency (*ω*), and *x* is the displacement in the x direction. The excitation force is the result of fluctuating electrostatic voltage from the high voltage comb drive pulse train and it can be derived from the rate of change of energy stored in the capacitor formed between a rotor finger and two stator fingers and is expressed as $F_{ext}=(n_{f}\varepsilon_{o}h)V_{pulse}^{2}/d_{gap}$, where *n_f_* is the total number of fingers, *ε_o_* is the vacuum permittivity, *h* is the height of the stator and rotor fingers, *d_gap_* is the gap between the stator and rotor fingers, and *V_pulse_* is the voltage pulse train output from the high voltage driver circuit. To preserve the linearity of the equation of motion, we neglect the sideways movement. The periodic voltage pulse train can be expressed as a Fourier series, as given by Equation (2).

(2) $$V(t)=V_{o}\left[\frac{\tau }{T}+\sum_{n=1}^{\infty }\frac{2}{n\pi }\sin\left(\frac{n\pi \tau }{T}\right)\cos\left(\frac{2n\pi }{T}\left(t-\frac{\tau }{2}\right)\right)\right]$$

where *V_o_* is the amplitude of voltage pulse, *τ* is the pulse width, and *T* is the time period which is equal to 2*π/ω*. The effective mass of the moving rotor can be estimated by applying the principle of conservation of energy on the resonating structure and assuming that all of beams deflect with mode shapes as if under static loads [16]. The energy conservation and beam theory together give the effective mass of the resonator as:

(3) $$m_{eff}=m_{s}+m_{f}+\frac{12}{35}m_{b}+\frac{1}{4}m_{t}$$

where *m_s_* is the mass of the shuttle, *m_f_* that of moving fingers, *m_b_* represents mass of eight parallel beams, and *m_t_* is the mass of two trusses. Assuming that the trusses act as rigid support for the beams under deflection, the total stiffness for the folded beams under no residual stress can be calculated by using the series and parallel beam theory. Since each beam length is not identical the effective stiffness along *x* axis becomes:

(4) $$k_{eff}=4\cdot \left(\frac{k_{x,b1}\cdot k_{x,b2}}{k_{x,b1}+k_{x,b2}}\right)$$

where *k_x,b_*_1_ and *k_x,b_*_2_ are the beam stiffness for beam elements *b*_1_ and *b*_2_, as shown in the figure. From Euler beam theory the stiffness of each beam segment is $k=12E_{Si}I_{z,b}/L^{3}$, where *E_Si_* is the Young’s modulus of silicon, *I_z,b_* is the area moment of inertia along the *z* axis, and *L* is length of the beam segment. The area moment of inertia for beam cross section is given by $I_{z,b}=hw^{3}/12$, where *h* is the height, *w* is the width of the beam. The material properties that were used to calculate the variables in the equation of motion are given in Table 1 below. Using these values, the effective mass of the rotor and the total stiffness for the folded beam arrangement is calculated to be 1.23 µg and 210 N/m, respectively which give the analytical resonant frequency $f_{n}=\sqrt{k_{eff}/m_{eff}}/2\pi$ as 65.7 kHz.

## 5. Experimental Imaging Results

Test comb drive microactuators were driven by a high voltage pulse train *V_COMB_* that was produced by the circuit of Figure 6a, and the device mechanical oscillation motion was captured by the microscope camera when illuminated by a stream of LED pulses that were strobed at a specific phase. The maximum comb drive voltage was kept below the side snap-in instability limit. The comb side-snap pull-in voltage [17] was calculated to be 300 V, but when corrected for dimensional errors in fabrication, it is close to 220 V.

### 5.1. Frequency Analysis

**Figure 10:**
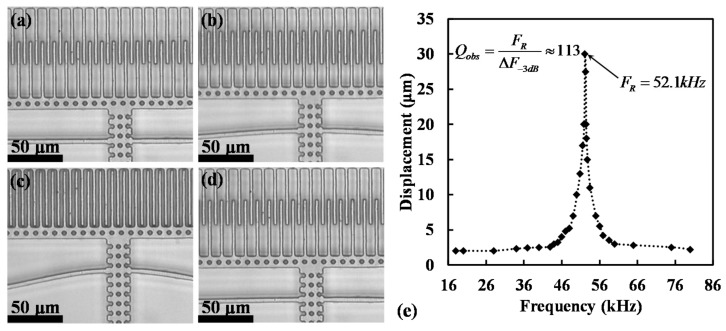
Frequency response through stroboscopic freeze frame images of comb drive actuator at maximum displacements for different frequencies (**a**) No actuation, (**b**) 50 kHz, (**c**) 52.1 kHz, (**d**) 80 kHz and (**e**) Frequency response graph for all of the frequencies tested giving observed *Q* factor ≈ 113 and resonance frequency of 52.1 kHz.

The comb actuators were driven with a voltage amplitude of 150 V over a wide frequency range. The device’s mechanical motion was strobe imaged at the excitation frequency at the phase that provides maximum displacement. The images were recorded at three frames per second (FPS). Using a low frame rate for recording gives longer light integration time, thus increasing image brightness and sharpness. Figure 10 shows the image of comb drive actuator frozen in motion at the maximum stroke for a few frequencies. The maximum stroke length data for 31 frequencies from 15 kHz to 80 kHz were plotted against the frequency. The experimental value of the quality factor can be calculated from this figure using the −3 dB frequency points in the graph. From this curve, we observed that the natural frequency of the comb drive actuator is 52.1 kHz, which is 20% off from the calculated resonant frequency. This error can be attributed to dimensional errors in the fabricated device and a trapezoidal profile of the DRIE etched features. The effect of these geometric errors has a pronounced impact on the beam stiffness and negligible effect on the effective mass of the system. We calculated the adjusted beam stiffness based on the observed resonant frequency as *k_adj_* = 132 N/m. The *Q* factor from the experimental result was 113, which when compared to the calculated analytical value (~155) as given in [18,19], is off by ~27%. Adjusting the *Q* factor value with the experimentally found *k_adj_* value gives a *Q* factor of 118, which is in good agreement with the observed value. The images at peak resonance show that the maximum displacement in *+x* direction was ~30 μm, so the total stroke *d_s_* of the resonating comb drive was ~60 μm.

### 5.2. Phase Analysis

**Figure 11:**
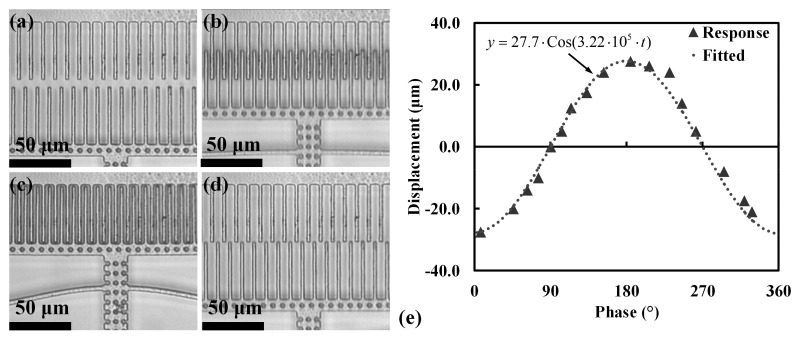
Phase resolved resonant motion of a test comb drive actuator imaged through stroboscopic imaging system at resonance at (**a**) 7°, (**b**) 90°, (**c**) 184°, (**d**) 330°, and (**e**) graph of displacement vs. phase w.r.t. the drive pulse shows a cosine relation between displacement and time, which is consistent with the device theory in discussed in Section 4.

The speed of motion $\upsilon =2\pi F_{R}\cdot d_{s}$ for this comb drive that was calculated from the images was ~20 m/s. Imaging at such high speed of motion requires a very sharp LED pulse of the order of ~50 ns. This was achieved by adjusting the R*C value in the LED driver circuit. With these sharp LED pulses, the resonance motion of the comb drive was imaged at different phases. Figure 11 shows the images that were captured by our stroboscopic system at different phases. The reference position of the shuttle without any actuation voltage is chosen as *x* = 0, and displacement for each phase of motion is approximated from the images w.r.t. the *x* = 0 position. This data was plotted, which showed that the motion follows a cosine curve, as was expected from analytical analysis.

### 5.3. Stroke vs. Applied Voltage

**Figure 12:**
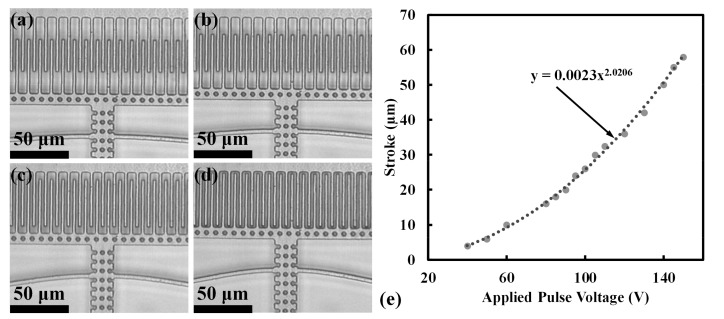
Effect of applied voltage to maximum displacement observed through stroboscopic imaging set-up at (**a**) 90 V, (**b**) 110 V, (**c**) 130 V, (**d**) 150 V, and (**e**) stroke is plotted against the applied voltage, and it was observed to have a square dependence on applied voltage which agrees with the device theory.

The effect of applied voltage to the maximum stroke was also observed, as shown in Figure 12. Note that the stroke length in the plot is twice the maximum displacement that was observed in the *+x* direction. The drive voltage was varied from 40 to 150 V. A square dependence of stroke length on the applied voltage is observed, which is consistent with the comb drive motion theory of Section 4.

### 5.4. Stroboscopic System Limitations

**Figure 13:**
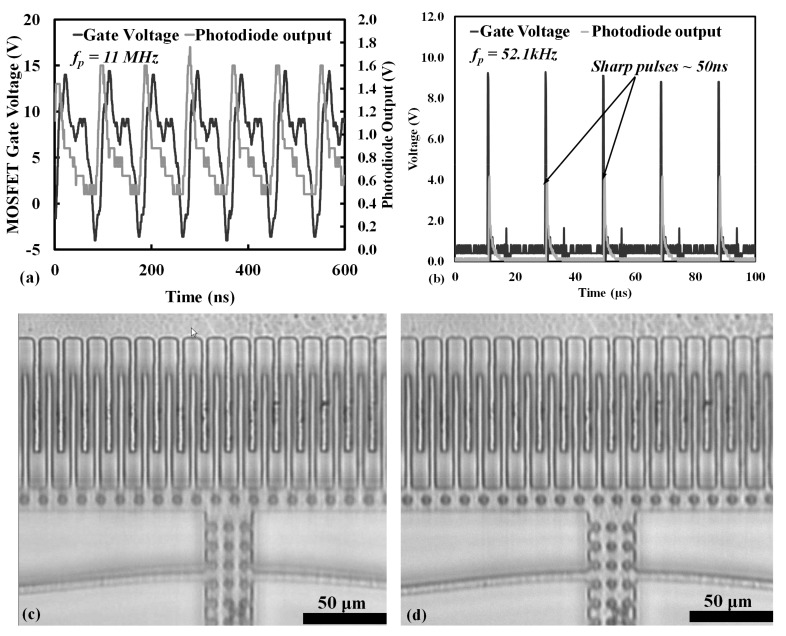
Performance limits of the imaging system. (**a**) The imaging system is shown to work well at srtobing frequencies up to 11 MHz frequency as shown by the pulsed LED output detected by a high-speed photodetector placed at the camera c-mount; (**b**) The sharpening of the LED pulses is shown at the resonant frequency of 52.1 kHz with 50 ns sharp pulses from an R*C differentiator; Figure (**c**) shows the motion blurring in the captured image at 52.1 kHz with rectangular 190 ns strobe pulse widths as achieved from a divide by 100 counter; (**d**) shows a reduction in motion blurring when the LED signal is sharpened with a differentiation circuit producing 50 ns wide strobe pulses.

The TTL timer generator implementation that is discussed here has a maximum input clock frequency of about 84 MHz [19], which at modulo factor *M* of 100 results in a maximum imaging frequency of 840 kHz, but other faster schemes are also possible using gate arrays or when operated a lower *M*. If a faster timer generator circuit is utilized, the fundamental limitation is determined by the maximum LED strobe frequency and pulse width. The maximum strobe frequency is limited by the maximum frequency at which the gate driver IC for the LED driver circuit, as shown in Figure 6b, can work stably. The pulsing of LED was captured on an avalanche photodetector (Thorlabs APD120A2/M—Si Avalanche Photodetector (Thorlabs, Newton, NJ, USA) that was connected to the oscilloscope. The LED pulse and the output from the photodetector was plotted together. The imaging system was observed to work flawlessly up to a strobe frequency of 11 MHz, as shown in the Figure 13a. Another constraint on the imaging system is determined by the width of the LED pulse that defines the phase interval of image exposure. A sharp pulse width ensures minimum blurring while capturing a dynamic MEMS actuator which results from a finite amount of movement of the micro-structure during the time for which the LED is on. The pulse width was controlled using an R*C differentiator circuit with a variable resistor such that a pulse width of 50–80 ns was maintained for all of the frequencies. It was observed that below 50 ns the LED failed turn on or provided very little light, making the frame rate of the camera very low.

The impact of the use of the R*C differentiator is shown in Figure 13b at a frequency of 52.1 kHz. Since we observed the speed at resonance to be ~20 m/s, an undifferentiated 190 ns LED on-time generated from the timing generator would results in an image blur of ~3.8 μm. However, when the pulse width is decreased to 50 ns with the differentiator, the motion blur of the comb-drive is reduced to ~1 μm, which is close to the optical resolution of the imaging system (0.6 μm). The difference in image clarity on the recorded images at the same phase of motion with and without the differentiator is shown in Figure 13c,d. Note that the differentiated LED signals produces a significant improvement in the image sharpness.

## 6. Conclusions

We have demonstrated the construction, testing, and performance limits of a low cost (USD < $2000) in-plane stroboscopic motion analyzer system. The system was evaluated using high speed comb drive microactuators. Resonant frequency of 52.1 kHz with a stroke length of 60 μm was observed. Resonant motion of the combs at velocities of 20 m/s was also successfully reconstructed from the imaging data. Small exposure times of ~50 ns and the utilization of low camera frame rates enabled the capturing of sharp and bright images at optical spatial resolution of 0.5 μm. No other stroboscopic imaging system has been previously reported to image microstructures at such high speeds and frequencies, making this imaging system an inexpensive alternative to commercially available imaging systems.

## Figures and Tables

**Table 1:** Dimensional and material properties for test comb drive devices.

Name	Parameter	Value	Unit
Shuttle length	*L_s_*	294	µm
Truss length	*L_t_*	153	µm
Width shuttle	*w_s_*	20	µm
Width truss	*w_t_*	10	µm
1st beam length	*L_b_*_1_	125	µm
2nd beam length	*L_b__2_*	90	µm
Beam width	*w_b_*	2.5	µm
Support length	*L_cs_*	293	µm
Support width	*w_cs_*	10	µm
Finger length	*L_f_*	50	µm
Finger width	*w_f_*	3	µm
Finger gap	*d_gap_*	2	µm
Finger overlap length	*L_o_*	22	µm
Number of rotor fingers	*N_fr_*	60	-
Number of stator fingers	*N_fs_*	58	-
Finger height	*h*	30	µm
Young’s modulus for silicon	*E_Si_*	150	GPa
Density of silicon	*ρ_Si_*	2330	Kg · m^−3^
Buffer oxide thickness	*d_SiO_2__*	1	µm
Kinematic viscosity of air	*ν_air_*	1.57 × 10^−5^	m^2^/s

## Supplementary Materials

The video is available online at www.mdpi.com/2072-666X/8/12/351/s1.
